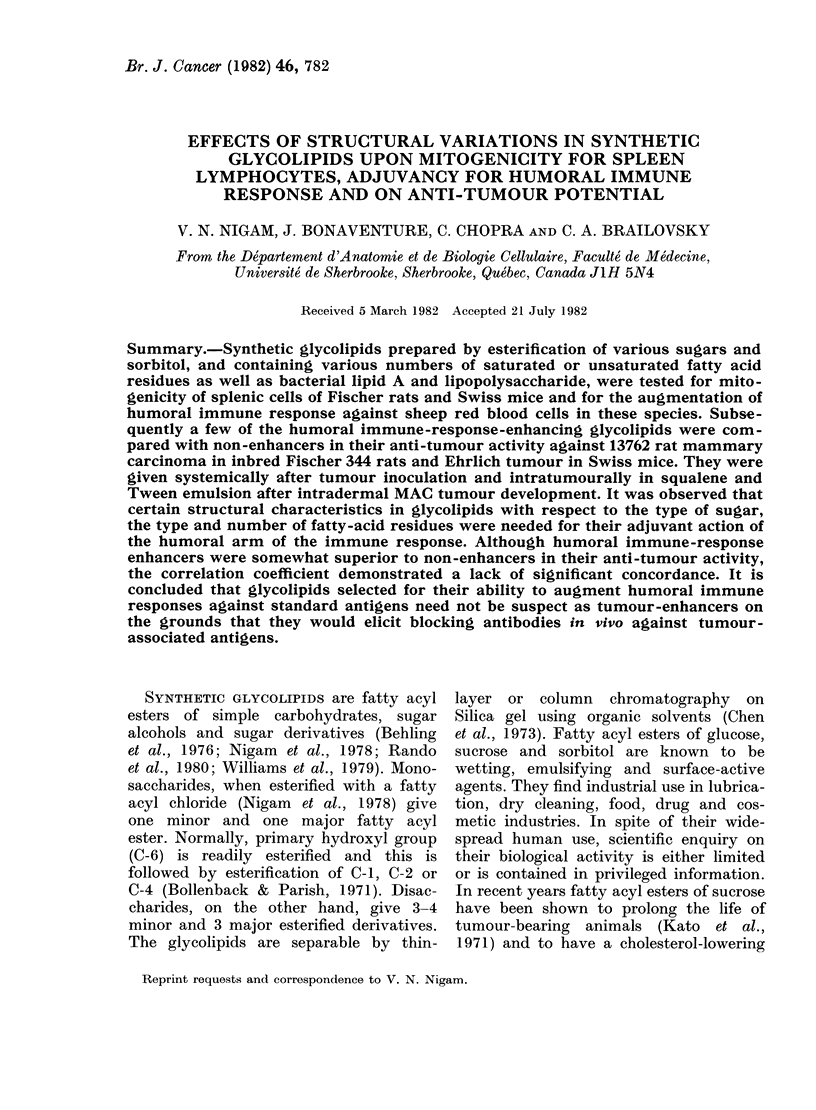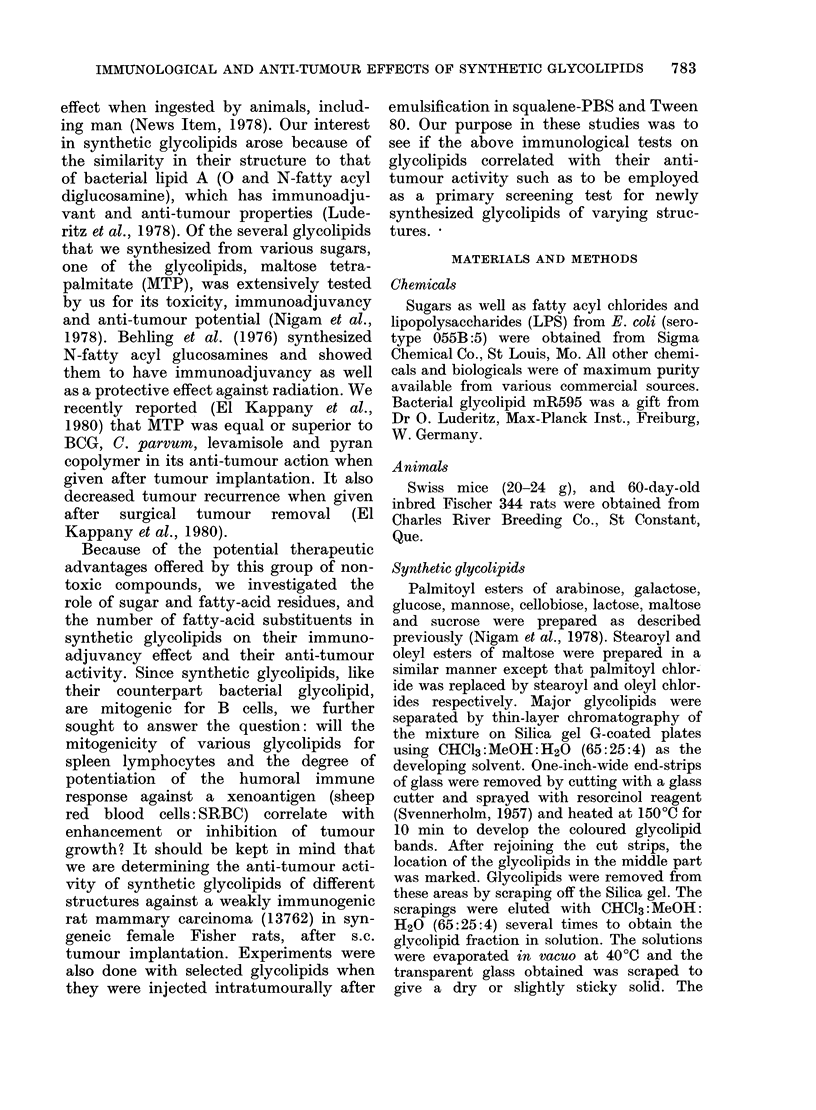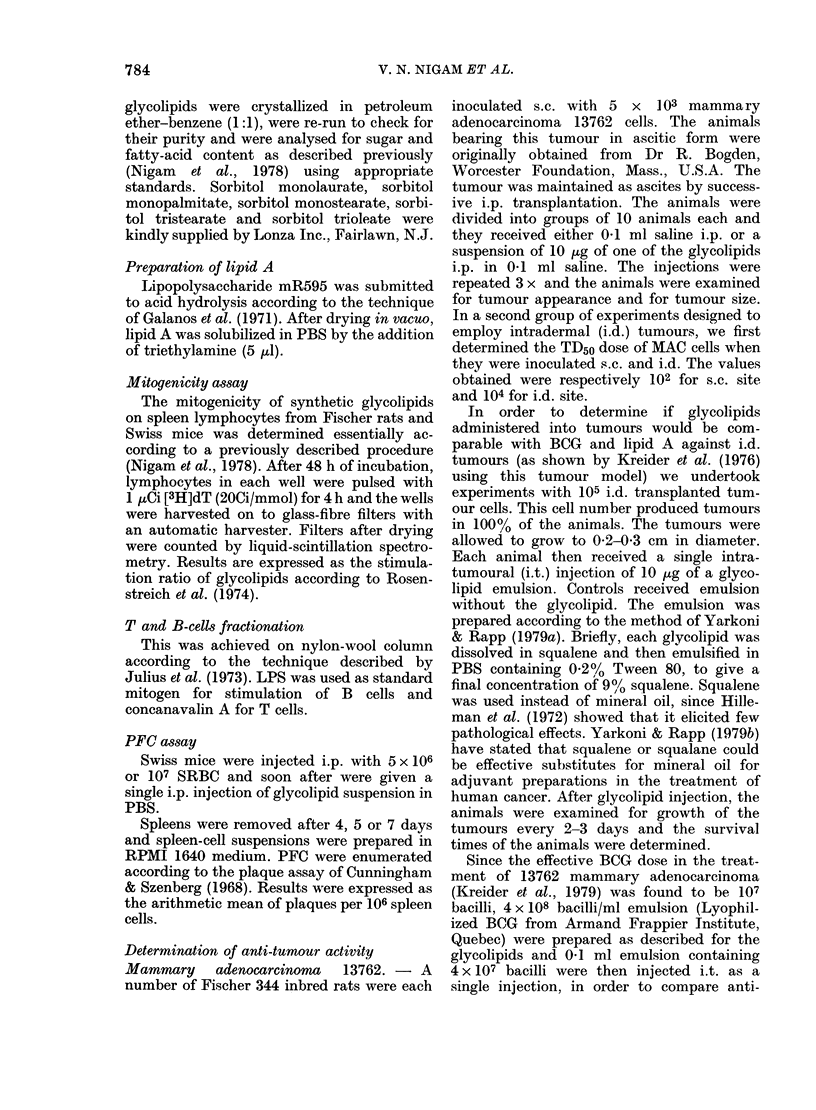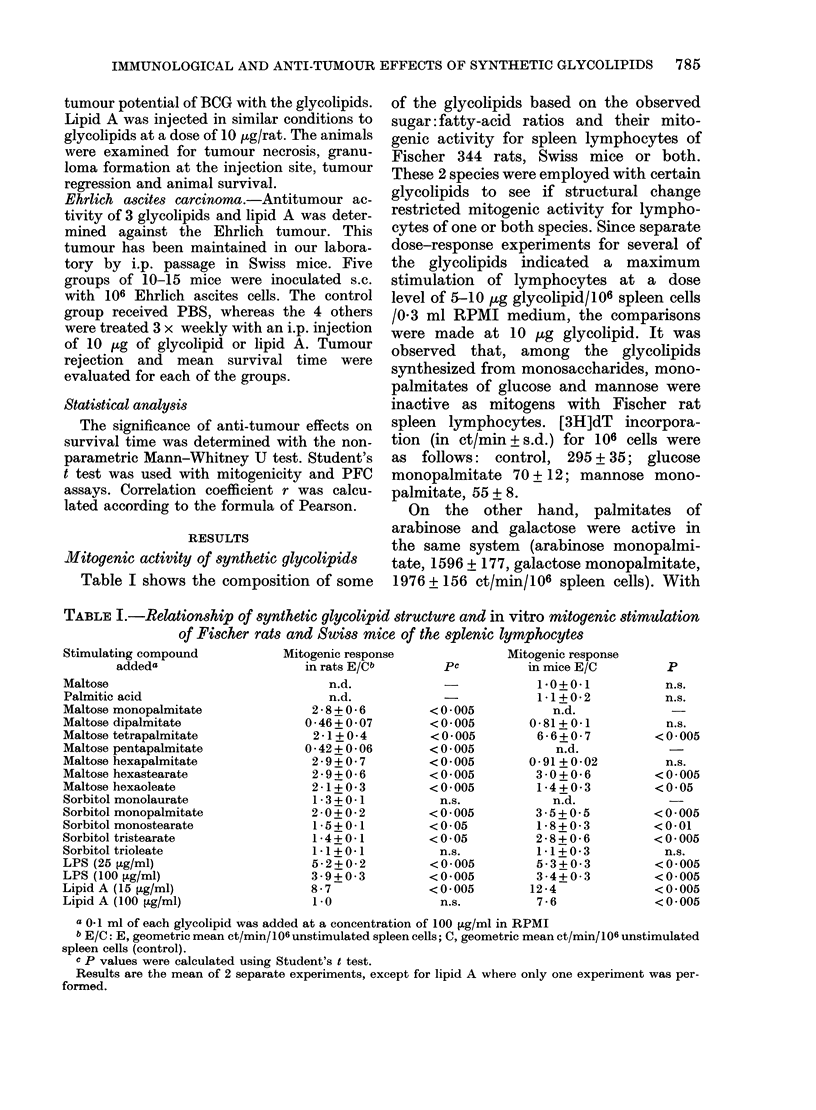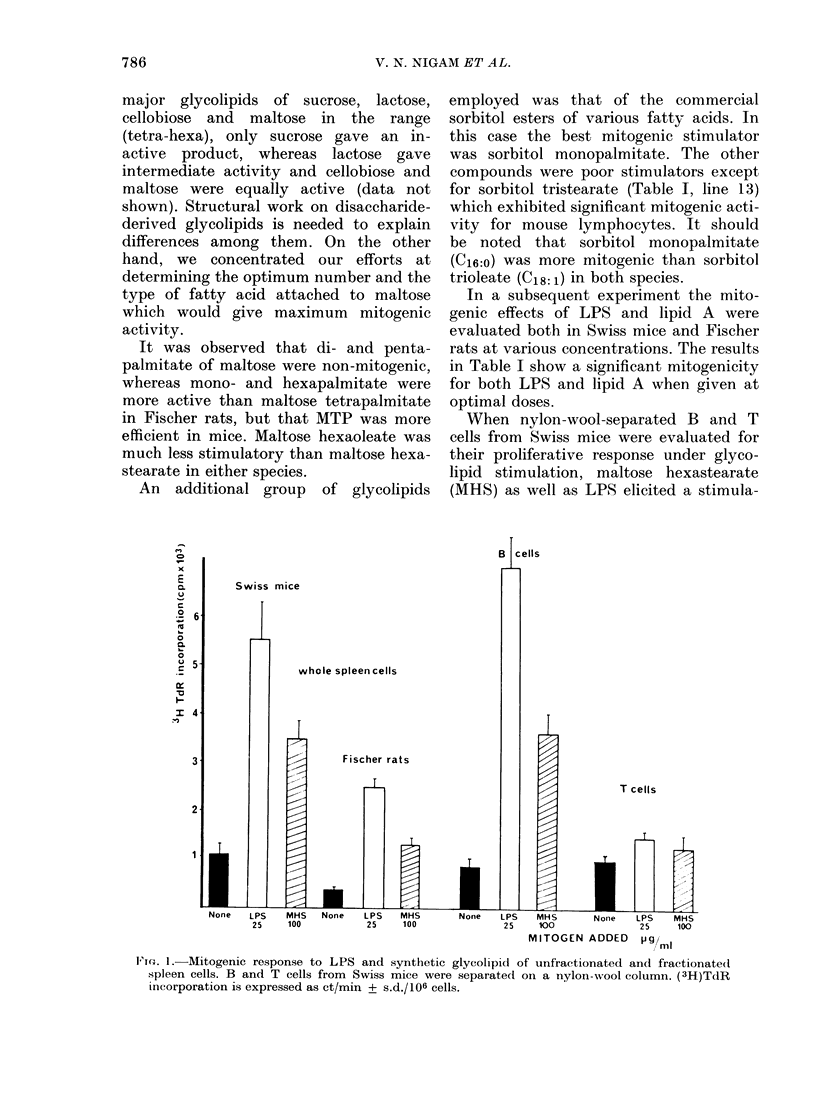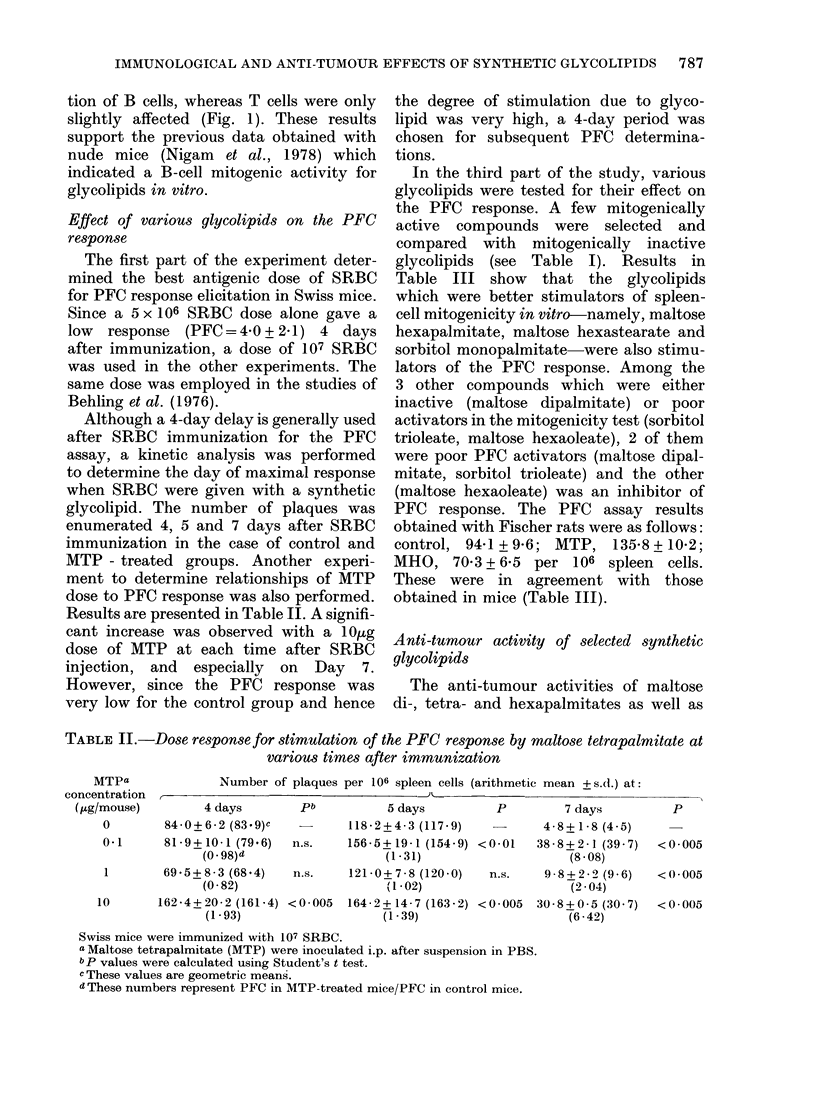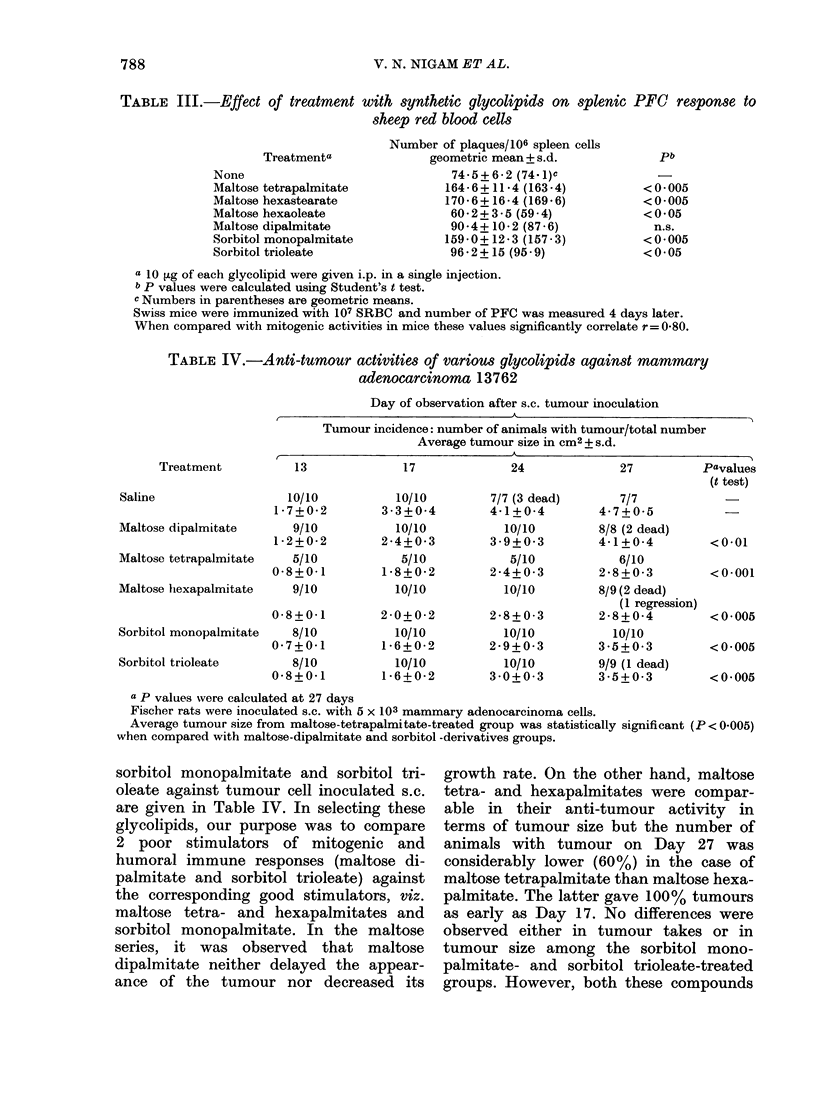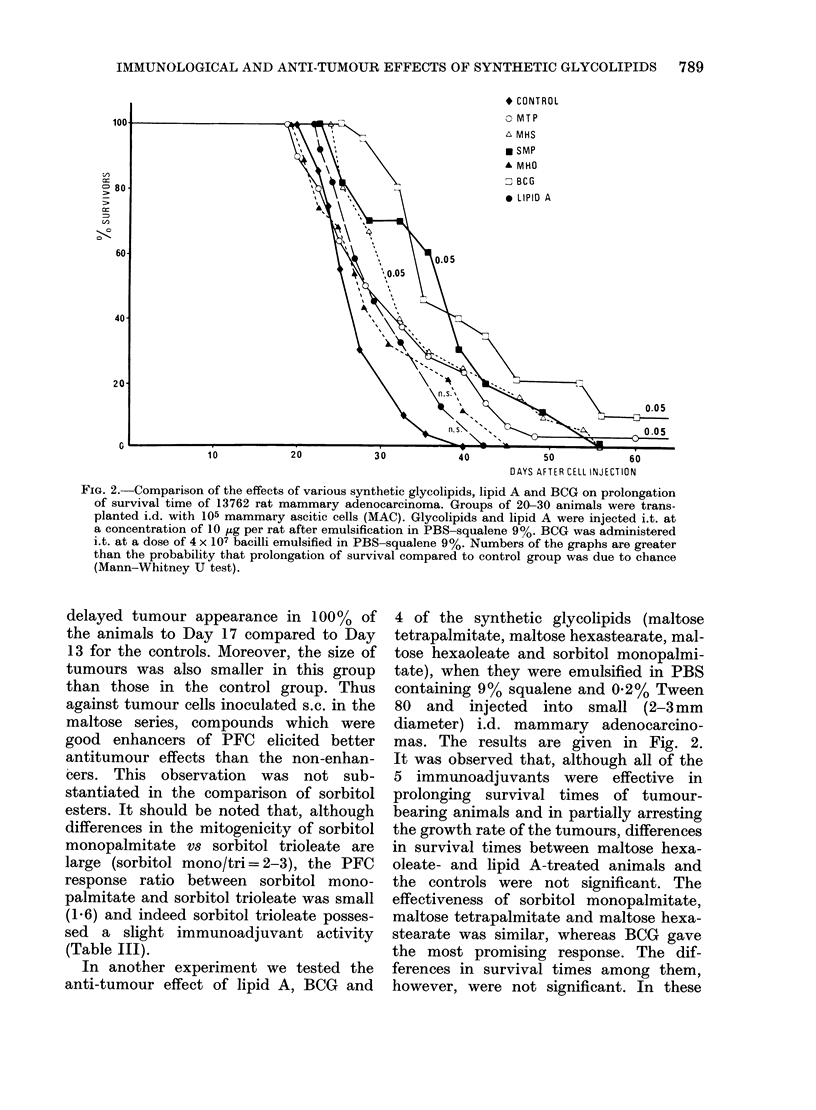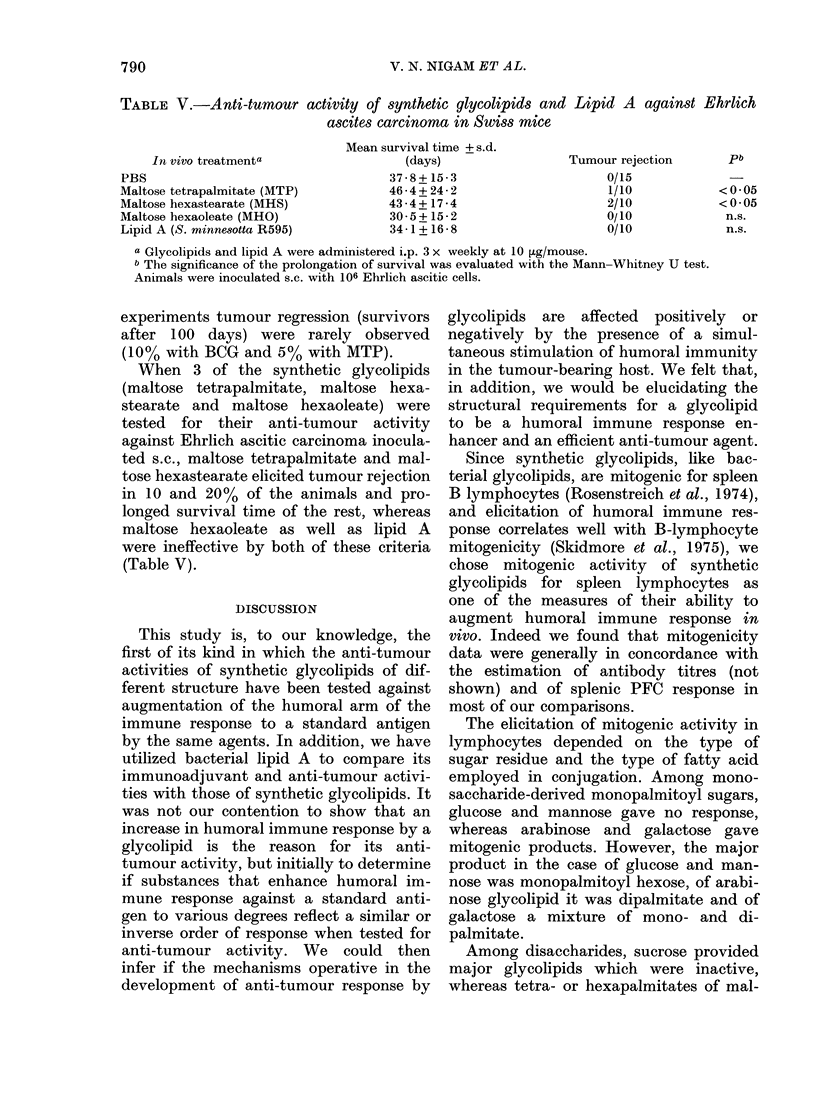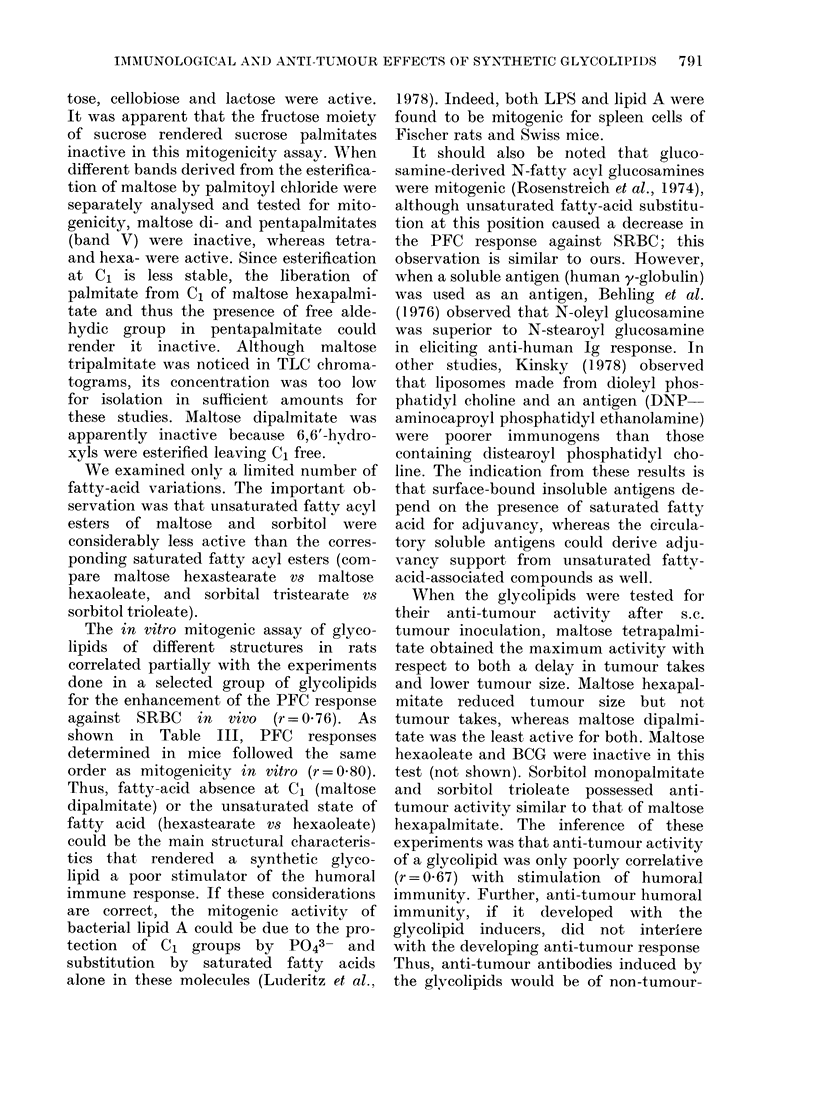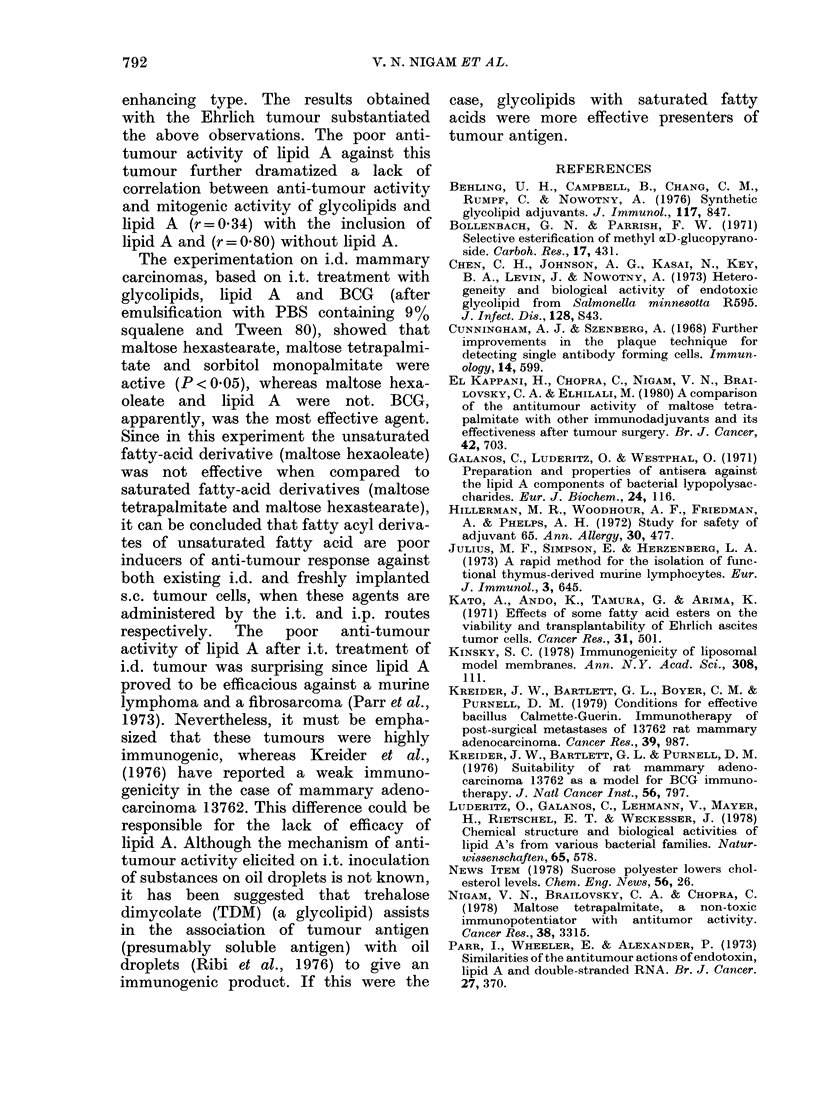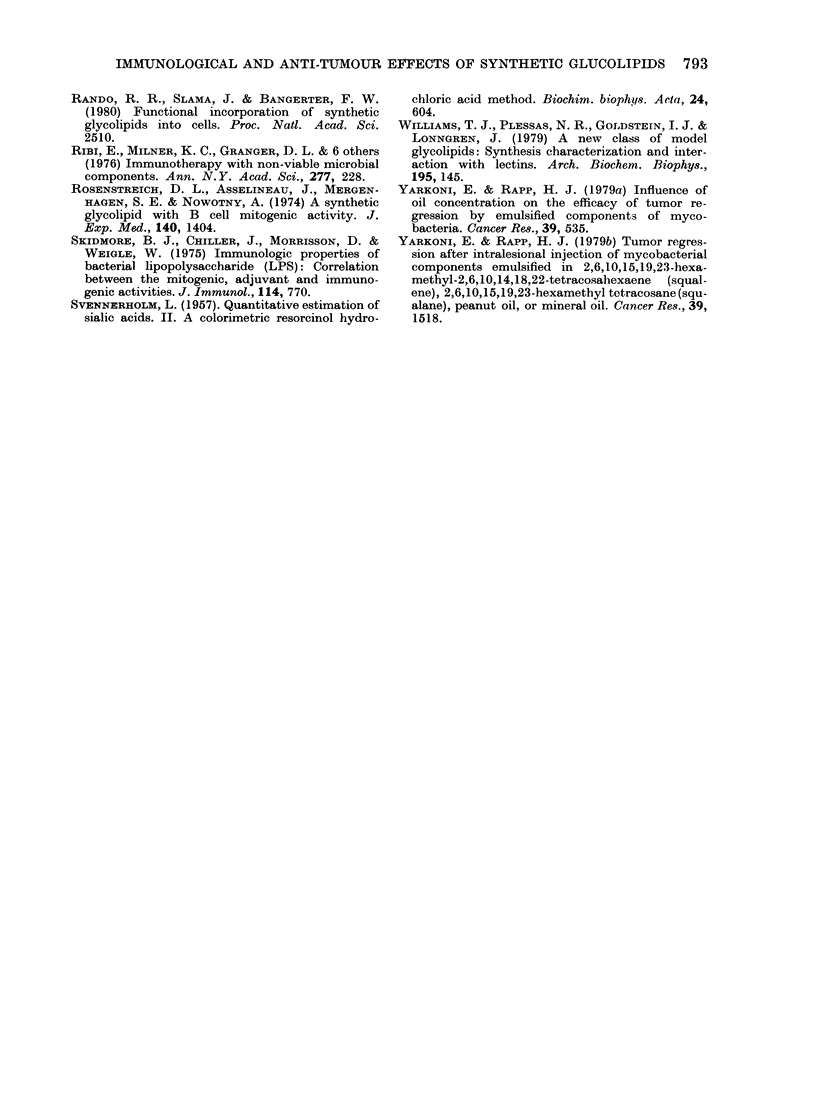# Effects of structural variations in synthetic glycolipids upon mitogenicity for spleen lymphocytes, adjuvancy for humoral immune response and on anti-tumour potential.

**DOI:** 10.1038/bjc.1982.271

**Published:** 1982-11

**Authors:** V. N. Nigam, J. Bonaventure, C. Chopra, C. A. Brailovsky

## Abstract

Synthetic glycolipids prepared by esterification of various sugars and sorbitol, and containing various numbers of saturated or unsaturated fatty acid residues as well as bacterial lipid A and lipopolysaccharide, were tested for mitogenicity of splenic cells of Fischer rats and Swiss mice and for the augmentation of humoral immune response against sheep red blood cells in these species. Subsequently a few of the humoral immune-response-enhancing glycolipids were compared with non-enhancers in their anti-tumour activity against 13762 rat mammary carcinoma in inbred Fischer 344 rats and Ehrlich tumour in Swiss mice. They were given systemically after tumour inoculation and intratumourally in squalene and Tween emulsion after intradermal MAC tumour development. It was observed that certain structural characteristics in glycolipids with respect to the type of sugar, the type and number of fatty-acid residues were needed for their adjuvant action of the humoral arm of the immune response. Although humoral immune-response enhancers were somewhat superior to non-enhancers in their anti-tumour activity, the correlation coefficient demonstrated a lack of significant concordance. It is concluded that glycolipids selected for their ability to augment humoral immune responses against standard antigens need not be suspect as tumour-enhancers on the grounds that they would elicit blocking antibodies in vivo against tumour-associated antigens.


*Br. J. Cancer* (1982) **46**, 782

### EFFECTS OF STRUCTURAL VARIATIONS IN SYNTHETIC GLYCOLIPIDS UPON MITOGENICITY FOR SPLEEN LYMPHOCYTES, ADJUVANCY FOR HUMORAL IMMUNE RESPONSE AND ON ANTI-TUMOUR POTENTIAL

V. N. NIGAM, J. BONAVENTURE, C. CHOPRA AND C. A. BRAILOVSKY

*From the Département d'Anatomie et de Biologie Cellulaire, Faculté de Médecine, Université de Sherbrooke, Sherbrooke, Québec, Canada J1H 5N4*

Received 5 March 1982 Accepted 21 July 1982

**Summary.—Synthetic glycolipids prepared by esterification of various sugars and sorbitol, and containing various numbers of saturated or unsaturated fatty acid residues as well as bacterial lipid A and lipopolysaccharide, were tested for mitogenicity of splenic cells of Fischer rats and Swiss mice and for the augmentation of humoral immune response against sheep red blood cells in these species. Subsequently a few of the humoral immune-response-enhancing glycolipids were compared with non-enhancers in their anti-tumour activity against 13762 rat mammary carcinoma in inbred Fischer 344 rats and Ehrlich tumour in Swiss mice. They were given systemically after tumour inoculation and intratumourally in squalene and Tween emulsion after intradermal MAC tumour development. It was observed that certain structural characteristics in glycolipids with respect to the type of sugar, the type and number of fatty-acid residues were needed for their adjuvant action of the humoral arm of the immune response. Although humoral immune-response enhancers were somewhat superior to non-enhancers in their anti-tumour activity, the correlation coefficient demonstrated a lack of significant concordance. It is concluded that glycolipids selected for their ability to augment humoral immune responses against standard antigens need not be suspect as tumour-enhancers on the grounds that they would elicit blocking antibodies *in vivo* against tumour-associated antigens.**

SYNTHETIC GLYCOLIPIDS are fatty acyl esters of simple carbohydrates, sugar alcohols and sugar derivatives (Behling *et al.*, 1976; Nigam *et al.*, 1978; Rando *et al.*, 1980; Williams *et al.*, 1979). Monosaccharides, when esterified with a fatty acyl chloride (Nigam *et al.*, 1978) give one minor and one major fatty acyl ester. Normally, primary hydroxyl group (C-6) is readily esterified and this is followed by esterification of C-1, C-2 or C-4 (Bollenback & Parish, 1971). Disaccharides, on the other hand, give 3–4 minor and 3 major esterified derivatives. The glycolipids are separable by thin-layer or column chromatography on Silica gel using organic solvents (Chen *et al.*, 1973). Fatty acyl esters of glucose, sucrose and sorbitol are known to be wetting, emulsifying and surface-active agents. They find industrial use in lubrication, dry cleaning, food, drug and cosmetic industries. In spite of their widespread human use, scientific enquiry on their biological activity is either limited or is contained in privileged information. In recent years fatty acyl esters of sucrose have been shown to prolong the life of tumour-bearing animals (Kato *et al.*, 1971) and to have a cholesterol-lowering

Reprint requests and correspondence to V. N. Nigam.

effect when ingested by animals, including man (News Item, 1978). Our interest in synthetic glycolipids arose because of the similarity in their structure to that of bacterial lipid A (O and N-fatty acyl diglucosamine), which has immunoadjuvant and anti-tumour properties (Luderitz *et al.*, 1978). Of the several glycolipids that we synthesized from various sugars, one of the glycolipids, maltose tetrapalmitate (MTP), was extensively tested by us for its toxicity, immunoadjuvancy and anti-tumour potential (Nigam *et al.*, 1978). Behling *et al.* (1976) synthesized N-fatty acyl glucosamines and showed them to have immunoadjuvancy as well as a protective effect against radiation. We recently reported (El Kappany *et al.*, 1980) that MTP was equal or superior to BCG, *C. parvum*, levamisole and pyran copolymer in its anti-tumour action when given after tumour implantation. It also decreased tumour recurrence when given after surgical tumour removal (El Kappany *et al.*, 1980).

Because of the potential therapeutic advantages offered by this group of non-toxic compounds, we investigated the role of sugar and fatty-acid residues, and the number of fatty-acid substituents in synthetic glycolipids on their immuno-adjuvancy effect and their anti-tumour activity. Since synthetic glycolipids, like their counterpart bacterial glycolipid, are mitogenic for B cells, we further sought to answer the question: will the mitogenicity of various glycolipids for spleen lymphocytes and the degree of potentiation of the humoral immune response against a xenoantigen (sheep red blood cells: SRBC) correlate with enhancement or inhibition of tumour growth? It should be kept in mind that we are determining the anti-tumour activity of synthetic glycolipids of different structures against a weakly immunogenic rat mammary carcinoma (13762) in syngeneic female Fisher rats, after s.c. tumour implantation. Experiments were also done with selected glycolipids when they were injected intratumourally after emulsification in squalene-PBS and Tween 80. Our purpose in these studies was to see if the above immunological tests on glycolipids correlated with their anti-tumour activity such as to be employed as a primary screening test for newly synthesized glycolipids of varying structures. ·

### MATERIALS AND METHODS

### *Chemicals*

Sugars as well as fatty acyl chlorides and lipopolysaccharides (LPS) from *E. coli* (serotype 055B:5) were obtained from Sigma Chemical Co., St Louis, Mo. All other chemicals and biologicals were of maximum purity available from various commercial sources. Bacterial glycolipid mR595 was a gift from Dr O. Luderitz, Max-Planck Inst., Freiburg, W. Germany.

### *Animals*

Swiss mice (20–24 g), and 60-day-old inbred Fischer 344 rats were obtained from Charles River Breeding Co., St Constant, Que.

### *Synthetic glycolipids*

Palmitoyl esters of arabinose, galactose, glucose, mannose, cellobiose, lactose, maltose and sucrose were prepared as described previously (Nigam *et al.*, 1978). Stearoyl and oleyl esters of maltose were prepared in a similar manner except that palmitoyl chloride was replaced by stearoyl and oleyl chlorides respectively. Major glycolipids were separated by thin-layer chromatography of the mixture on Silica gel G-coated plates using $CHCl_3$:MeOH:$H_2O$ (65:25:4) as the developing solvent. One-inch-wide end-strips of glass were removed by cutting with a glass cutter and sprayed with resorcinol reagent (Svennerholm, 1957) and heated at 150°C for 10 min to develop the coloured glycolipid bands. After rejoining the cut strips, the location of the glycolipids in the middle part was marked. Glycolipids were removed from these areas by scraping off the Silica gel. The scrapings were eluted with $CHCl_3$:MeOH:$H_2O$ (65:25:4) several times to obtain the glycolipid fraction in solution. The solutions were evaporated *in vacuo* at 40°C and the transparent glass obtained was scraped to give a dry or slightly sticky solid. The glycolipids were crystallized in petroleum ether–benzene (1:1), were re-run to check for their purity and were analysed for sugar and fatty-acid content as described previously (Nigam *et al.*, 1978) using appropriate standards. Sorbitol monolaurate, sorbitol monopalmitate, sorbitol monostearate, sorbitol tristearate and sorbitol trioleate were kindly supplied by Lonza Inc., Fairlawn, N.J.

*Preparation of lipid A*

Lipopolysaccharide mR595 was submitted to acid hydrolysis according to the technique of Galanos *et al.* (1971). After drying *in vacuo*, lipid A was solubilized in PBS by the addition of triethylamine (5 $\mu$l).

*Mitogenicity assay*

The mitogenicity of synthetic glycolipids on spleen lymphocytes from Fischer rats and Swiss mice was determined essentially according to a previously described procedure (Nigam *et al.*, 1978). After 48 h of incubation, lymphocytes in each well were pulsed with 1 $\mu$Ci [$^3$H]dT (20Ci/mmol) for 4 h and the wells were harvested on to glass-fibre filters with an automatic harvester. Filters after drying were counted by liquid-scintillation spectrometry. Results are expressed as the stimulation ratio of glycolipids according to Rosenstreich *et al.* (1974).

*T and B-cells fractionation*

This was achieved on nylon-wool column according to the technique described by Julius *et al.* (1973). LPS was used as standard mitogen for stimulation of B cells and concanavalin A for T cells.

*PFC assay*

Swiss mice were injected i.p. with $5 \times 10^6$ or $10^7$ SRBC and soon after were given a single i.p. injection of glycolipid suspension in PBS.

Spleens were removed after 4, 5 or 7 days and spleen-cell suspensions were prepared in RPMI 1640 medium. PFC were enumerated according to the plaque assay of Cunningham & Szenberg (1968). Results were expressed as the arithmetic mean of plaques per $10^6$ spleen cells.

*Determination of anti-tumour activity*

*Mammary adenocarcinoma* 13762. — A number of Fischer 344 inbred rats were each inoculated s.c. with $5 \times 10^3$ mammary adenocarcinoma 13762 cells. The animals bearing this tumour in ascitic form were originally obtained from Dr R. Bogden, Worcester Foundation, Mass., U.S.A. The tumour was maintained as ascites by successive i.p. transplantation. The animals were divided into groups of 10 animals each and they received either 0·1 ml saline i.p. or a suspension of 10 $\mu$g of one of the glycolipids i.p. in 0·1 ml saline. The injections were repeated 3× and the animals were examined for tumour appearance and for tumour size. In a second group of experiments designed to employ intradermal (i.d.) tumours, we first determined the $TD_{50}$ dose of MAC cells when they were inoculated s.c. and i.d. The values obtained were respectively $10^2$ for s.c. site and $10^4$ for i.d. site.

In order to determine if glycolipids administered into tumours would be comparable with BCG and lipid A against i.d. tumours (as shown by Kreider *et al.* (1976) using this tumour model) we undertook experiments with $10^5$ i.d. transplanted tumour cells. This cell number produced tumours in 100% of the animals. The tumours were allowed to grow to 0·2–0·3 cm in diameter. Each animal then received a single intratumoural (i.t.) injection of 10 $\mu$g of a glycolipid emulsion. Controls received emulsion without the glycolipid. The emulsion was prepared according to the method of Yarkoni & Rapp (1979*a*). Briefly, each glycolipid was dissolved in squalene and then emulsified in PBS containing 0·2% Tween 80, to give a final concentration of 9% squalene. Squalene was used instead of mineral oil, since Hilleman *et al.* (1972) showed that it elicited few pathological effects. Yarkoni & Rapp (1979*b*) have stated that squalene or squalane could be effective substitutes for mineral oil for adjuvant preparations in the treatment of human cancer. After glycolipid injection, the animals were examined for growth of the tumours every 2–3 days and the survival times of the animals were determined.

Since the effective BCG dose in the treatment of 13762 mammary adenocarcinoma (Kreider *et al.*, 1979) was found to be $10^7$ bacilli, $4 \times 10^8$ bacilli/ml emulsion (Lyophilized BCG from Armand Frappier Institute, Quebec) were prepared as described for the glycolipids and 0·1 ml emulsion containing $4 \times 10^7$ bacilli were then injected i.t. as a single injection, in order to compare antitumour potential of BCG with the glycolipids. Lipid A was injected in similar conditions to glycolipids at a dose of 10 μg/rat. The animals were examined for tumour necrosis, granuloma formation at the injection site, tumour regression and animal survival.

*Ehrlich ascites carcinoma.*—Antitumour activity of 3 glycolipids and lipid A was determined against the Ehrlich tumour. This tumour has been maintained in our laboratory by i.p. passage in Swiss mice. Five groups of 10–15 mice were inoculated s.c. with $10^6$ Ehrlich ascites cells. The control group received PBS, whereas the 4 others were treated 3 × weekly with an i.p. injection of 10 μg of glycolipid or lipid A. Tumour rejection and mean survival time were evaluated for each of the groups.

### *Statistical analysis*

The significance of anti-tumour effects on survival time was determined with the non-parametric Mann–Whitney U test. Student's *t* test was used with mitogenicity and PFC assays. Correlation coefficient *r* was calculated according to the formula of Pearson.

### RESULTS

### *Mitogenic activity of synthetic glycolipids*

Table I shows the composition of some of the glycolipids based on the observed sugar:fatty-acid ratios and their mitogenic activity for spleen lymphocytes of Fischer 344 rats, Swiss mice or both. These 2 species were employed with certain glycolipids to see if structural change restricted mitogenic activity for lymphocytes of one or both species. Since separate dose–response experiments for several of the glycolipids indicated a maximum stimulation of lymphocytes at a dose level of 5–10 μg glycolipid/$10^6$ spleen cells /0·3 ml RPMI medium, the comparisons were made at 10 μg glycolipid. It was observed that, among the glycolipids synthesized from monosaccharides, monopalmitates of glucose and mannose were inactive as mitogens with Fischer rat spleen lymphocytes. [3H]dT incorporation (in ct/min ± s.d.) for $10^6$ cells were as follows: control, 295 ± 35; glucose monopalmitate 70 ± 12; mannose monopalmitate, 55 ± 8.

On the other hand, palmitates of arabinose and galactose were active in the same system (arabinose monopalmitate, 1596 ± 177, galactose monopalmitate, 1976 ± 156 ct/min/$10^6$ spleen cells). With

TABLE I.—*Relationship of synthetic glycolipid structure and* in vitro *mitogenic stimulation of Fischer rats and Swiss mice of the splenic lymphocytes*

| Stimulating compound added[a] | Mitogenic response in rats E/C[b] | P[c] | Mitogenic response in mice E/C | P |
|---|---|---|---|---|
| Maltose | n.d. | — | 1·0 ± 0·1 | n.s. |
| Palmitic acid | n.d. | — | 1·1 ± 0·2 | n.s. |
| Maltose monopalmitate | 2·8 ± 0·6 | < 0·005 | n.d. | — |
| Maltose dipalmitate | 0·46 ± 0·07 | < 0·005 | 0·81 ± 0·1 | n.s. |
| Maltose tetrapalmitate | 2·1 ± 0·4 | < 0·005 | 6·6 ± 0·7 | < 0·005 |
| Maltose pentapalmitate | 0·42 ± 0·06 | < 0·005 | n.d. | — |
| Maltose hexapalmitate | 2·9 ± 0·7 | < 0·005 | 0·91 ± 0·02 | n.s. |
| Maltose hexastearate | 2·9 ± 0·6 | < 0·005 | 3·0 ± 0·6 | < 0·005 |
| Maltose hexaoleate | 2·1 ± 0·3 | < 0·005 | 1·4 ± 0·3 | < 0·05 |
| Sorbitol monolaurate | 1·3 ± 0·1 | n.s. | n.d. | — |
| Sorbitol monopalmitate | 2·0 ± 0·2 | < 0·005 | 3·5 ± 0·5 | < 0·005 |
| Sorbitol monostearate | 1·5 ± 0·1 | < 0·05 | 1·8 ± 0·3 | < 0·01 |
| Sorbitol tristearate | 1·4 ± 0·1 | < 0·05 | 2·8 ± 0·6 | < 0·005 |
| Sorbitol trioleate | 1·1 ± 0·1 | n.s. | 1·1 ± 0·3 | n.s. |
| LPS (25 μg/ml) | 5·2 ± 0·2 | < 0·005 | 5·3 ± 0·3 | < 0·005 |
| LPS (100 μg/ml) | 3·9 ± 0·3 | < 0·005 | 3·4 ± 0·3 | < 0·005 |
| Lipid A (15 μg/ml) | 8·7 | < 0·005 | 12·4 | < 0·005 |
| Lipid A (100 μg/ml) | 1·0 | n.s. | 7·6 | < 0·005 |

[a] 0·1 ml of each glycolipid was added at a concentration of 100 μg/ml in RPMI

[b] E/C: E, geometric mean ct/min/$10^6$ unstimulated spleen cells; C, geometric mean ct/min/$10^6$ unstimulated spleen cells (control).

[c] *P* values were calculated using Student's *t* test.

Results are the mean of 2 separate experiments, except for lipid A where only one experiment was performed.

major glycolipids of sucrose, lactose, cellobiose and maltose in the range (tetra-hexa), only sucrose gave an inactive product, whereas lactose gave intermediate activity and cellobiose and maltose were equally active (data not shown). Structural work on disaccharide-derived glycolipids is needed to explain differences among them. On the other hand, we concentrated our efforts at determining the optimum number and the type of fatty acid attached to maltose which would give maximum mitogenic activity.

It was observed that di- and penta-palmitate of maltose were non-mitogenic, whereas mono- and hexapalmitate were more active than maltose tetrapalmitate in Fischer rats, but that MTP was more efficient in mice. Maltose hexaoleate was much less stimulatory than maltose hexastearate in either species.

An additional group of glycolipids employed was that of the commercial sorbitol esters of various fatty acids. In this case the best mitogenic stimulator was sorbitol monopalmitate. The other compounds were poor stimulators except for sorbitol tristearate (Table I, line 13) which exhibited significant mitogenic activity for mouse lymphocytes. It should be noted that sorbitol monopalmitate ($C_{16:0}$) was more mitogenic than sorbitol trioleate ($C_{18:1}$) in both species.

In a subsequent experiment the mitogenic effects of LPS and lipid A were evaluated both in Swiss mice and Fischer rats at various concentrations. The results in Table I show a significant mitogenicity for both LPS and lipid A when given at optimal doses.

When nylon-wool-separated B and T cells from Swiss mice were evaluated for their proliferative response under glycolipid stimulation, maltose hexastearate (MHS) as well as LPS elicited a stimula-

FIG. 1.—Mitogenic response to LPS and synthetic glycolipid of unfractionated and fractionated spleen cells. B and T cells from Swiss mice were separated on a nylon-wool column. ($^3$H)TdR incorporation is expressed as ct/min ± s.d./$10^6$ cells.

tion of B cells, whereas T cells were only slightly affected (Fig. 1). These results support the previous data obtained with nude mice (Nigam *et al.*, 1978) which indicated a B-cell mitogenic activity for glycolipids *in vitro*.

### *Effect of various glycolipids on the PFC response*

The first part of the experiment determined the best antigenic dose of SRBC for PFC response elicitation in Swiss mice. Since a $5 \times 10^6$ SRBC dose alone gave a low response (PFC = $4{\cdot}0 \pm 2{\cdot}1$) 4 days after immunization, a dose of $10^7$ SRBC was used in the other experiments. The same dose was employed in the studies of Behling *et al.* (1976).

Although a 4-day delay is generally used after SRBC immunization for the PFC assay, a kinetic analysis was performed to determine the day of maximal response when SRBC were given with a synthetic glycolipid. The number of plaques was enumerated 4, 5 and 7 days after SRBC immunization in the case of control and MTP - treated groups. Another experiment to determine relationships of MTP dose to PFC response was also performed. Results are presented in Table II. A significant increase was observed with a 10μg dose of MTP at each time after SRBC injection, and especially on Day 7. However, since the PFC response was very low for the control group and hence the degree of stimulation due to glycolipid was very high, a 4-day period was chosen for subsequent PFC determinations.

In the third part of the study, various glycolipids were tested for their effect on the PFC response. A few mitogenically active compounds were selected and compared with mitogenically inactive glycolipids (see Table I). Results in Table III show that the glycolipids which were better stimulators of spleen-cell mitogenicity *in vitro*—namely, maltose hexapalmitate, maltose hexastearate and sorbitol monopalmitate—were also stimulators of the PFC response. Among the 3 other compounds which were either inactive (maltose dipalmitate) or poor activators in the mitogenicity test (sorbitol trioleate, maltose hexaoleate), 2 of them were poor PFC activators (maltose dipalmitate, sorbitol trioleate) and the other (maltose hexaoleate) was an inhibitor of PFC response. The PFC assay results obtained with Fischer rats were as follows: control, $94{\cdot}1 \pm 9{\cdot}6$; MTP, $135{\cdot}8 \pm 10{\cdot}2$; MHO, $70{\cdot}3 \pm 6{\cdot}5$ per $10^6$ spleen cells. These were in agreement with those obtained in mice (Table III).

### *Anti-tumour activity of selected synthetic glycolipids*

The anti-tumour activities of maltose di-, tetra- and hexapalmitates as well as

TABLE II.—*Dose response for stimulation of the PFC response by maltose tetrapalmitate at various times after immunization*

| MTP[a] concentration (μg/mouse) | Number of plaques per $10^6$ spleen cells (arithmetic mean ± s.d.) at: 4 days | $P^b$ | 5 days | $P$ | 7 days | $P$ |
|---|---|---|---|---|---|---|
| 0 | 84·0±6·2 (83·9)[c] | — | 118·2±4·3 (117·9) | — | 4·8±1·8 (4·5) | — |
| 0·1 | 81·9±10·1 (79·6) (0·98)[d] | n.s. | 156·5±19·1 (154·9) (1·31) | <0·01 | 38·8±2·1 (39·7) (8·08) | <0·005 |
| 1 | 69·5±8·3 (68·4) (0·82) | n.s. | 121·0±7·8 (120·0) (1·02) | n.s. | 9·8±2·2 (9·6) (2·04) | <0·005 |
| 10 | 162·4±20·2 (161·4) (1·93) | <0·005 | 164·2±14·7 (163·2) (1·39) | <0·005 | 30·8±0·5 (30·7) (6·42) | <0·005 |

Swiss mice were immunized with $10^7$ SRBC.
[a] Maltose tetrapalmitate (MTP) were inoculated i.p. after suspension in PBS.
[b] *P* values were calculated using Student's *t* test.
[c] These values are geometric means.
[d] These numbers represent PFC in MTP-treated mice/PFC in control mice.

TABLE III.—*Effect of treatment with synthetic glycolipids on splenic PFC response to sheep red blood cells*

| Treatment[a] | Number of plaques/$10^6$ spleen cells geometric mean ± s.d. | $P^b$ |
|---|---|---|
| None | 74·5 ± 6·2 (74·1)[c] | — |
| Maltose tetrapalmitate | 164·6 ± 11·4 (163·4) | <0·005 |
| Maltose hexastearate | 170·6 ± 16·4 (169·6) | <0·005 |
| Maltose hexaoleate | 60·2 ± 3·5 (59·4) | <0·05 |
| Maltose dipalmitate | 90·4 ± 10·2 (87·6) | n.s. |
| Sorbitol monopalmitate | 159·0 ± 12·3 (157·3) | <0·005 |
| Sorbitol trioleate | 96·2 ± 15 (95·9) | <0·05 |

[a] 10 μg of each glycolipid were given i.p. in a single injection.
[b] *P* values were calculated using Student's *t* test.
[c] Numbers in parentheses are geometric means.
Swiss mice were immunized with $10^7$ SRBC and number of PFC was measured 4 days later.
When compared with mitogenic activities in mice these values significantly correlate $r = 0{\cdot}80$.

TABLE IV.—*Anti-tumour activities of various glycolipids against mammary adenocarcinoma* 13762

| Treatment | Day of observation after s.c. tumour inoculation. Tumour incidence: number of animals with tumour/total number; Average tumour size in cm² ± s.d. — 13 | 17 | 24 | 27 | $P^a$ values ($t$ test) |
|---|---|---|---|---|---|
| Saline | 10/10 | 10/10 | 7/7 (3 dead) | 7/7 | — |
| | 1·7 ± 0·2 | 3·3 ± 0·4 | 4·1 ± 0·4 | 4·7 ± 0·5 | — |
| Maltose dipalmitate | 9/10 | 10/10 | 10/10 | 8/8 (2 dead) | |
| | 1·2 ± 0·2 | 2·4 ± 0·3 | 3·9 ± 0·3 | 4·1 ± 0·4 | <0·01 |
| Maltose tetrapalmitate | 5/10 | 5/10 | 5/10 | 6/10 | |
| | 0·8 ± 0·1 | 1·8 ± 0·2 | 2·4 ± 0·3 | 2·8 ± 0·3 | <0·001 |
| Maltose hexapalmitate | 9/10 | 10/10 | 10/10 | 8/9 (2 dead) (1 regression) | |
| | 0·8 ± 0·1 | 2·0 ± 0·2 | 2·8 ± 0·3 | 2·8 ± 0·4 | <0·005 |
| Sorbitol monopalmitate | 8/10 | 10/10 | 10/10 | 10/10 | |
| | 0·7 ± 0·1 | 1·6 ± 0·2 | 2·9 ± 0·3 | 3·5 ± 0·3 | <0·005 |
| Sorbitol trioleate | 8/10 | 10/10 | 10/10 | 9/9 (1 dead) | |
| | 0·8 ± 0·1 | 1·6 ± 0·2 | 3·0 ± 0·3 | 3·5 ± 0·3 | <0·005 |

[a] *P* values were calculated at 27 days
Fischer rats were inoculated s.c. with $5 \times 10^3$ mammary adenocarcinoma cells.
Average tumour size from maltose-tetrapalmitate-treated group was statistically significant ($P < 0{\cdot}005$) when compared with maltose-dipalmitate and sorbitol -derivatives groups.

sorbitol monopalmitate and sorbitol trioleate against tumour cell inoculated s.c. are given in Table IV. In selecting these glycolipids, our purpose was to compare 2 poor stimulators of mitogenic and humoral immune responses (maltose dipalmitate and sorbitol trioleate) against the corresponding good stimulators, *viz.* maltose tetra- and hexapalmitates and sorbitol monopalmitate. In the maltose series, it was observed that maltose dipalmitate neither delayed the appearance of the tumour nor decreased its growth rate. On the other hand, maltose tetra- and hexapalmitates were comparable in their anti-tumour activity in terms of tumour size but the number of animals with tumour on Day 27 was considerably lower (60%) in the case of maltose tetrapalmitate than maltose hexapalmitate. The latter gave 100% tumours as early as Day 17. No differences were observed either in tumour takes or in tumour size among the sorbitol monopalmitate- and sorbitol trioleate-treated groups. However, both these compounds

FIG. 2.—Comparison of the effects of various synthetic glycolipids, lipid A and BCG on prolongation of survival time of 13762 rat mammary adenocarcinoma. Groups of 20–30 animals were transplanted i.d. with $10^5$ mammary ascitic cells (MAC). Glycolipids and lipid A were injected i.t. at a concentration of 10 $\mu$g per rat after emulsification in PBS–squalene 9%. BCG was administered i.t. at a dose of $4 \times 10^7$ bacilli emulsified in PBS–squalene 9%. Numbers of the graphs are greater than the probability that prolongation of survival compared to control group was due to chance (Mann–Whitney U test).

delayed tumour appearance in 100% of the animals to Day 17 compared to Day 13 for the controls. Moreover, the size of tumours was also smaller in this group than those in the control group. Thus against tumour cells inoculated s.c. in the maltose series, compounds which were good enhancers of PFC elicited better antitumour effects than the non-enhancers. This observation was not substantiated in the comparison of sorbitol esters. It should be noted that, although differences in the mitogenicity of sorbitol monopalmitate *vs* sorbitol trioleate are large (sorbitol mono/tri = 2–3), the PFC response ratio between sorbitol monopalmitate and sorbitol trioleate was small (1·6) and indeed sorbitol trioleate possessed a slight immunoadjuvant activity (Table III).

In another experiment we tested the anti-tumour effect of lipid A, BCG and 4 of the synthetic glycolipids (maltose tetrapalmitate, maltose hexastearate, maltose hexaoleate and sorbitol monopalmitate), when they were emulsified in PBS containing 9% squalene and 0·2% Tween 80 and injected into small (2–3 mm diameter) i.d. mammary adenocarcinomas. The results are given in Fig. 2. It was observed that, although all of the 5 immunoadjuvants were effective in prolonging survival times of tumour-bearing animals and in partially arresting the growth rate of the tumours, differences in survival times between maltose hexaoleate- and lipid A-treated animals and the controls were not significant. The effectiveness of sorbitol monopalmitate, maltose tetrapalmitate and maltose hexastearate was similar, whereas BCG gave the most promising response. The differences in survival times among them, however, were not significant. In these

TABLE V.—*Anti-tumour activity of synthetic glycolipids and Lipid A against Ehrlich ascites carcinoma in Swiss mice*

| *In vivo* treatment[a] | Mean survival time ± s.d. (days) | Tumour rejection | $P^b$ |
|---|---|---|---|
| PBS | 37·8 ± 15·3 | 0/15 | — |
| Maltose tetrapalmitate (MTP) | 46·4 ± 24·2 | 1/10 | < 0·05 |
| Maltose hexastearate (MHS) | 43·4 ± 17·4 | 2/10 | < 0·05 |
| Maltose hexaoleate (MHO) | 30·5 ± 15·2 | 0/10 | n.s. |
| Lipid A (*S. minnesotta* R595) | 34·1 ± 16·8 | 0/10 | n.s. |

[a] Glycolipids and lipid A were administered i.p. 3 × weekly at 10 µg/mouse.
[b] The significance of the prolongation of survival was evaluated with the Mann–Whitney U test.
Animals were inoculated s.c. with $10^6$ Ehrlich ascitic cells.

experiments tumour regression (survivors after 100 days) were rarely observed (10% with BCG and 5% with MTP).

When 3 of the synthetic glycolipids (maltose tetrapalmitate, maltose hexastearate and maltose hexaoleate) were tested for their anti-tumour activity against Ehrlich ascitic carcinoma inoculated s.c., maltose tetrapalmitate and maltose hexastearate elicited tumour rejection in 10 and 20% of the animals and prolonged survival time of the rest, whereas maltose hexaoleate as well as lipid A were ineffective by both of these criteria (Table V).

### DISCUSSION

This study is, to our knowledge, the first of its kind in which the anti-tumour activities of synthetic glycolipids of different structure have been tested against augmentation of the humoral arm of the immune response to a standard antigen by the same agents. In addition, we have utilized bacterial lipid A to compare its immunoadjuvant and anti-tumour activities with those of synthetic glycolipids. It was not our contention to show that an increase in humoral immune response by a glycolipid is the reason for its anti-tumour activity, but initially to determine if substances that enhance humoral immune response against a standard antigen to various degrees reflect a similar or inverse order of response when tested for anti-tumour activity. We could then infer if the mechanisms operative in the development of anti-tumour response by glycolipids are affected positively or negatively by the presence of a simultaneous stimulation of humoral immunity in the tumour-bearing host. We felt that, in addition, we would be elucidating the structural requirements for a glycolipid to be a humoral immune response enhancer and an efficient anti-tumour agent.

Since synthetic glycolipids, like bacterial glycolipids, are mitogenic for spleen B lymphocytes (Rosenstreich *et al.*, 1974), and elicitation of humoral immune response correlates well with B-lymphocyte mitogenicity (Skidmore *et al.*, 1975), we chose mitogenic activity of synthetic glycolipids for spleen lymphocytes as one of the measures of their ability to augment humoral immune response *in vivo*. Indeed we found that mitogenicity data were generally in concordance with the estimation of antibody titres (not shown) and of splenic PFC response in most of our comparisons.

The elicitation of mitogenic activity in lymphocytes depended on the type of sugar residue and the type of fatty acid employed in conjugation. Among monosaccharide-derived monopalmitoyl sugars, glucose and mannose gave no response, whereas arabinose and galactose gave mitogenic products. However, the major product in the case of glucose and mannose was monopalmitoyl hexose, of arabinose glycolipid it was dipalmitate and of galactose a mixture of mono- and dipalmitate.

Among disaccharides, sucrose provided major glycolipids which were inactive, whereas tetra- or hexapalmitates of maltose, cellobiose and lactose were active. It was apparent that the fructose moiety of sucrose rendered sucrose palmitates inactive in this mitogenicity assay. When different bands derived from the esterification of maltose by palmitoyl chloride were separately analysed and tested for mitogenicity, maltose di- and pentapalmitates (band V) were inactive, whereas tetra- and hexa- were active. Since esterification at $C_1$ is less stable, the liberation of palmitate from $C_1$ of maltose hexapalmitate and thus the presence of free aldehydic group in pentapalmitate could render it inactive. Although maltose tripalmitate was noticed in TLC chromatograms, its concentration was too low for isolation in sufficient amounts for these studies. Maltose dipalmitate was apparently inactive because 6,6′-hydroxyls were esterified leaving $C_1$ free.

We examined only a limited number of fatty-acid variations. The important observation was that unsaturated fatty acyl esters of maltose and sorbitol were considerably less active than the corresponding saturated fatty acyl esters (compare maltose hexastearate *vs* maltose hexaoleate, and sorbital tristearate *vs* sorbitol trioleate).

The *in vitro* mitogenic assay of glycolipids of different structures in rats correlated partially with the experiments done in a selected group of glycolipids for the enhancement of the PFC response against SRBC *in vivo* ($r = 0{\cdot}76$). As shown in Table III, PFC responses determined in mice followed the same order as mitogenicity *in vitro* ($r = 0{\cdot}80$). Thus, fatty-acid absence at $C_1$ (maltose dipalmitate) or the unsaturated state of fatty acid (hexastearate *vs* hexaoleate) could be the main structural characteristics that rendered a synthetic glycolipid a poor stimulator of the humoral immune response. If these considerations are correct, the mitogenic activity of bacterial lipid A could be due to the protection of $C_1$ groups by $PO_4^{3-}$ and substitution by saturated fatty acids alone in these molecules (Luderitz *et al.*, 1978). Indeed, both LPS and lipid A were found to be mitogenic for spleen cells of Fischer rats and Swiss mice.

It should also be noted that glucosamine-derived N-fatty acyl glucosamines were mitogenic (Rosenstreich *et al.*, 1974), although unsaturated fatty-acid substitution at this position caused a decrease in the PFC response against SRBC; this observation is similar to ours. However, when a soluble antigen (human $\gamma$-globulin) was used as an antigen, Behling *et al.* (1976) observed that N-oleyl glucosamine was superior to N-stearoyl glucosamine in eliciting anti-human Ig response. In other studies, Kinsky (1978) observed that liposomes made from dioleyl phosphatidyl choline and an antigen (DNP—aminocaproyl phosphatidyl ethanolamine) were poorer immunogens than those containing distearoyl phosphatidyl choline. The indication from these results is that surface-bound insoluble antigens depend on the presence of saturated fatty acid for adjuvancy, whereas the circulatory soluble antigens could derive adjuvancy support from unsaturated fatty-acid-associated compounds as well.

When the glycolipids were tested for their anti-tumour activity after s.c. tumour inoculation, maltose tetrapalmitate obtained the maximum activity with respect to both a delay in tumour takes and lower tumour size. Maltose hexapalmitate reduced tumour size but not tumour takes, whereas maltose dipalmitate was the least active for both. Maltose hexaoleate and BCG were inactive in this test (not shown). Sorbitol monopalmitate and sorbitol trioleate possessed anti-tumour activity similar to that of maltose hexapalmitate. The inference of these experiments was that anti-tumour activity of a glycolipid was only poorly correlative ($r = 0{\cdot}67$) with stimulation of humoral immunity. Further, anti-tumour humoral immunity, if it developed with the glycolipid inducers, did not interfere with the developing anti-tumour response Thus, anti-tumour antibodies induced by the glycolipids would be of non-tumourenhancing type. The results obtained with the Ehrlich tumour substantiated the above observations. The poor anti-tumour activity of lipid A against this tumour further dramatized a lack of correlation between anti-tumour activity and mitogenic activity of glycolipids and lipid A ($r = 0{\cdot}34$) with the inclusion of lipid A and ($r = 0{\cdot}80$) without lipid A.

The experimentation on i.d. mammary carcinomas, based on i.t. treatment with glycolipids, lipid A and BCG (after emulsification with PBS containing 9% squalene and Tween 80), showed that maltose hexastearate, maltose tetrapalmitate and sorbitol monopalmitate were active ($P < 0{\cdot}05$), whereas maltose hexaoleate and lipid A were not. BCG, apparently, was the most effective agent. Since in this experiment the unsaturated fatty-acid derivative (maltose hexaoleate) was not effective when compared to saturated fatty-acid derivatives (maltose tetrapalmitate and maltose hexastearate), it can be concluded that fatty acyl derivates of unsaturated fatty acid are poor inducers of anti-tumour response against both existing i.d. and freshly implanted s.c. tumour cells, when these agents are administered by the i.t. and i.p. routes respectively. The poor anti-tumour activity of lipid A after i.t. treatment of i.d. tumour was surprising since lipid A proved to be efficacious against a murine lymphoma and a fibrosarcoma (Parr *et al.*, 1973). Nevertheless, it must be emphasized that these tumours were highly immunogenic, whereas Kreider *et al.*, (1976) have reported a weak immunogenicity in the case of mammary adenocarcinoma 13762. This difference could be responsible for the lack of efficacy of lipid A. Although the mechanism of anti-tumour activity elicited on i.t. inoculation of substances on oil droplets is not known, it has been suggested that trehalose dimycolate (TDM) (a glycolipid) assists in the association of tumour antigen (presumably soluble antigen) with oil droplets (Ribi *et al.*, 1976) to give an immunogenic product. If this were the case, glycolipids with saturated fatty acids were more effective presenters of tumour antigen.

### REFERENCES

BEHLING, U. H., CAMPBELL, B., CHANG, C. M., RUMPF, C. & NOWOTNY, A. (1976) Synthetic glycolipid adjuvants. *J. Immunol.*, **117**, 847.

BOLLENBACH, G. N. & PARRISH, F. W. (1971) Selective esterification of methyl αD-glucopyranoside. *Carboh. Res.*, **17**, 431.

CHEN, C. H., JOHNSON, A. G., KASAI, N., KEY, B. A., LEVIN, J. & NOWOTNY, A. (1973) Heterogeneity and biological activity of endotoxic glycolipid from *Salmonella minnesotta* R595. *J. Infect. Dis.*, **128**, S43.

CUNNINGHAM, A. J. & SZENBERG, A. (1968) Further improvements in the plaque technique for detecting single antibody forming cells. *Immunology*, **14**, 599.

EL KAPPANI, H., CHOPRA, C., NIGAM, V. N., BRAILOVSKY, C. A. & ELHILALI, M. (1980) A comparison of the antitumour activity of maltose tetrapalmitate with other immunodadjuvants and its effectiveness after tumour surgery. *Br. J. Cancer*, **42**, 703.

GALANOS, C., LUDERITZ, O. & WESTPHAL, O. (1971) Preparation and properties of antisera against the lipid A components of bacterial lypopolysaccharides. *Eur. J. Biochem.*, **24**, 116.

HILLERMAN, M. R., WOODHOUR, A. F., FRIEDMAN, A. & PHELPS, A. H. (1972) Study for safety of adjuvant 65. *Ann. Allergy*, **30**, 477.

JULIUS, M. F., SIMPSON, E. & HERZENBERG, L. A. (1973) A rapid method for the isolation of functional thymus-derived murine lymphocytes. *Eur. J. Immunol.*, **3**, 645.

KATO, A., ANDO, K., TAMURA, G. & ARIMA, K. (1971) Effects of some fatty acid esters on the viability and transplantability of Ehrlich ascites tumor cells. *Cancer Res.*, **31**, 501.

KINSKY, S. C. (1978) Immunogenicity of liposomal model membranes. *Ann. N.Y. Acad. Sci.*, **308**, 111.

KREIDER, J. W., BARTLETT, G. L., BOYER, C. M. & PURNELL, D. M. (1979) Conditions for effective bacillus Calmette-Guerin. Immunotherapy of post-surgical metastases of 13762 rat mammary adenocarcinoma. *Cancer Res.*, **39**, 987.

KREIDER, J. W., BARTLETT, G. L. & PURNELL, D. M. (1976) Suitability of rat mammary adenocarcinoma 13762 as a model for BCG immunotherapy. *J. Natl Cancer Inst.*, **56**, 797.

LUDERITZ, O., GALANOS, C., LEHMANN, V., MAYER, H., RIETSCHEL, E. T. & WECKESSER, J. (1978) Chemical structure and biological activities of lipid A's from various bacterial families. *Naturwissenschaften*, **65**, 578.

NEWS ITEM (1978) Sucrose polyester lowers cholesterol levels. *Chem. Eng. News*, **56**, 26.

NIGAM, V. N., BRAILOVSKY, C. A. & CHOPRA, C. (1978) Maltose tetrapalmitate, a non-toxic immunopotentiator with antitumor activity. *Cancer Res.*, **38**, 3315.

PARR, I., WHEELER, E. & ALEXANDER, P. (1973) Similarities of the antitumour actions of endotoxin, lipid A and double-stranded RNA. *Br. J. Cancer*. **27**, 370.

RANDO, R. R., SLAMA, J. & BANGERTER, F. W. (1980) Functional incorporation of synthetic glycolipids into cells. *Proc. Natl. Acad. Sci.* 2510.

RIBI, E., MILNER, K. C., GRANGER, D. L. & 6 others (1976) Immunotherapy with non-viable microbial components. *Ann. N.Y. Acad. Sci.*, **277**, 228.

ROSENSTREICH, D. L., ASSELINEAU, J., MERGENHAGEN, S. E. & NOWOTNY, A. (1974) A synthetic glycolipid with B cell mitogenic activity. *J. Exp. Med.*, **140**, 1404.

SKIDMORE, B. J., CHILLER, J., MORRISSON, D. & WEIGLE, W. (1975) Immunologic properties of bacterial lipopolysaccharide (LPS): Correlation between the mitogenic, adjuvant and immunogenic activities. *J. Immunol.*, **114**, 770.

SVENNERHOLM, L. (1957). Quantitative estimation of sialic acids. II. A colorimetric resorcinol hydrochloric acid method. *Biochim. biophys. Acta*, **24**, 604.

WILLIAMS, T. J., PLESSAS, N. R., GOLDSTEIN, I. J. & LONNGREN, J. (1979) A new class of model glycolipids: Synthesis characterization and interaction with lectins. *Arch. Biochem. Biophys.*, **195**, 145.

YARKONI, E. & RAPP, H. J. (1979*a*) Influence of oil concentration on the efficacy of tumor regression by emulsified components of mycobacteria. *Cancer Res.*, **39**, 535.

YARKONI, E. & RAPP, H. J. (1979*b*) Tumor regression after intralesional injection of mycobacterial components emulsified in 2,6,10,15,19,23-hexamethyl-2,6,10,14,18,22-tetracosahexaene (squalene), 2,6,10,15,19,23-hexamethyl tetracosane (squalane), peanut oil, or mineral oil. *Cancer Res.*, **39**, 1518.